# Development of Therapeutic-Grade Small Interfering RNAs by Chemical Engineering

**DOI:** 10.3389/fgene.2012.00154

**Published:** 2012-08-20

**Authors:** Jesper B. Bramsen, Jørgen Kjems

**Affiliations:** ^1^Interdisciplinary Nanoscience Center, Department of Molecular Biology and Genetics, Aarhus UniversityAarhus C, Denmark

**Keywords:** RNAi, siRNA, chemical modification, immunogenicity, off-target effect, LNA, OMe, siRNA therapeutic

## Abstract

Recent successes in clinical trials have provided important proof of concept that small interfering RNAs (siRNAs) indeed constitute a new promising class of therapeutics. Although great efforts are still needed to ensure efficient means of delivery *in vivo*, the siRNA molecule itself has been successfully engineered by chemical modification to meet initial challenges regarding specificity, stability, and immunogenicity. To date, a great wealth of siRNA architectures and types of chemical modification are available for promoting safe siRNA-mediated gene silencing *in vivo* and, consequently, the choice of design and modification types can be challenging to individual experimenters. Here we review the literature and devise how to improve siRNA performance by structural design and specific chemical modification to ensure potent and specific gene silencing without unwarranted side-effects and hereby complement the ongoing efforts to improve cell targeting and delivery by other carrier molecules.

## Silencing Genes Using Nucleic Acid

### Nucleic acid-based therapeutics

Nucleic acid-based therapeutics promise to overcome the major limitation of existing medicine, which can currently only target a limited number of proteins involved in disease pathways (Melnikova, 2007). Such promise rely on the high predictability of nucleic acid base-pairing which provides an ideal framework for gene silencing technologies (GSTs) by offering unparalleled specificity, rapidity of development and renders, at least in principle, all human genes “druggable”(Krieg, 2011). Pioneering work in the 1970–1980s established the nucleic acid antisense technology as an universal GST by developing synthetic antisense oligonucleotides (ASOs) and ribozymes, which base pair to and inhibit the function of any desired messenger RNA (mRNA; Zamecnik and Stephenson, 1978; Potera, 2007). Today, two ASO-based drugs have been commercialized and several modern antisense design variants (Monia et al., 1993; Highleyman, 1998; Elmen et al., 2008; Gupta et al., 2010; Cirak et al., 2011) may be on the verge of success with >50 RNA or RNA-derived therapeutics reaching clinical testing (Sanghvi, 2011; Burnett and Rossi, 2012). This journey has, however, been far from straightforward and tedious efforts have been invested to engineer poorly performing drug candidates such as first generation ASO designs by chemical modification (Stein and Krieg, 1994) to meet therapeutic standards of potency and safety (Potera, 2007).

### Exploiting RNAi pathways for therapeutics

**Figure 1:**
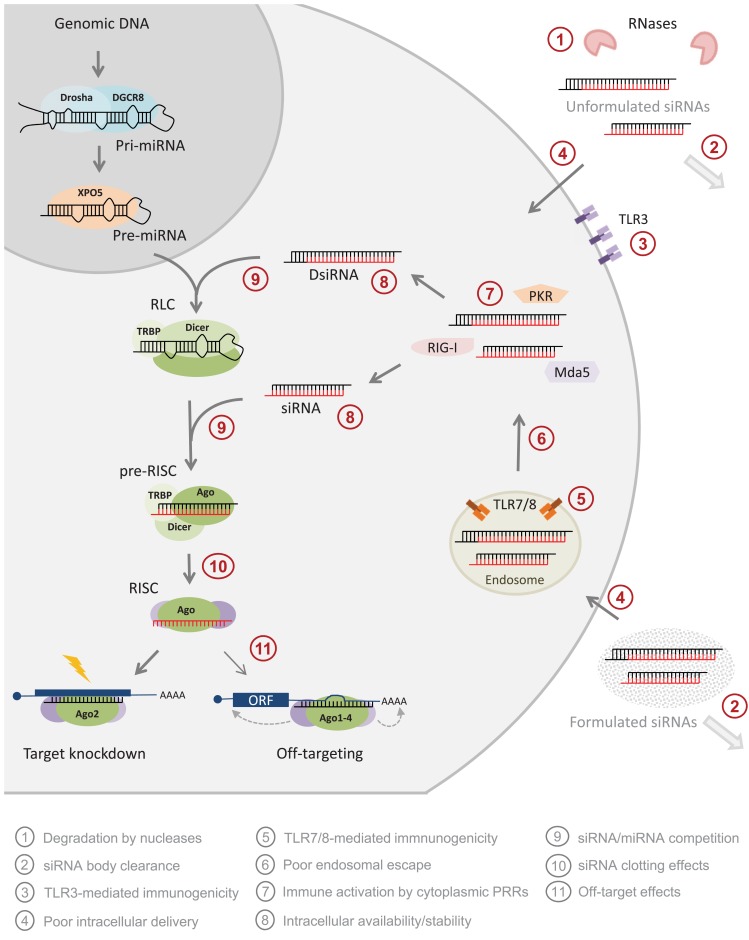
**The benefits and limitations of synthetic siRNA application**. The most widely used siRNA type is the “canonical” synthetic 21-mer siRNA composed of two 21 nt RNA strands annealed to form a 19-bp dsRNA duplex stem and 2 nt 3′-overhangs at both ends (the passenger strand is shown in black and the guide strand is shown in red). Also synthetic, dicer-substrate 27-mer siRNAs (DsiRNA) has provided a popular alternative. Both design types can be delivered *in vivo* either unformulated or upon formulation of various types of delivery agents into the cell cytoplasm (light gray circle) where siRNAs are taken up by a RISC loading complex (RLC), which upon a dicer cleavage event (27-mer designs only, 21-mer siRNAs are dicer-independent) is structurally rearranged into a pre-RISC. Here the siRNA passenger strand is cleaved leading to the establishment of an active RISC that assists and ensures efficient degradation of RNA target sharing perfect sequence complementarity to the siRNA guide stands. A number of bottleneck in siRNA applications are currently being resolved by chemical modification strategies (red circles). Unformulated siRNAs are sensitive to nuclease degradation in extracellular environment 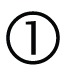 and, although degradation rates are much lower in the cytoplasm, siRNAs stabilization by modification is suggested to also enhance intracellular availability and silencing persistence 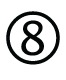. Also, extracellular siRNAs can be rapidly cleared from the body, e.g., by renal filtration 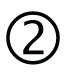 and can induce innate immune responses via TLR3 in certain cells 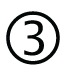. Delivery across the target cell membrane 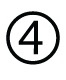 and endosomal release of endocytosed 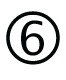 are currently the main bottlenecks in siRNA applications *in vivo* and siRNAs may induce TLR7/8-mediated immune-responses upon endosome retention in immune cells 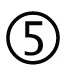. Also, all cells can respond to foreign cytoplasmic RNA via the PRRs, PKR, RIG-I, and Mda5 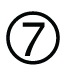. siRNA may disturb natural miRNA pathways, that processes nuclear pri-miRNA transcripts (dark gray circle) via a pre-miRNA intermediate and miRNA duplex into a single-stranded miRNA in RISC, by direct competition for RISC loading 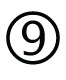 or by clotting the pathway due to slow siRNA processing and turnover 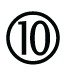. Finally, all siRNA will trigger miRNA-like off-targeting effects on unintended targets upon base-pairing of the guide strand seed region and positions within mRNA 3′ UTRs leading to transcript destabilization and/or translational repression 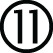. Please refer to main text for more detail.

The discovery of RNAi interference (RNAi), gene silencing by double-stranded RNA (dsRNA), in the nematode worm *C. elegans* in 1998 (Fire et al., 1998) and the observation in 2001 that synthetic 21-mer dsRNA, named small interfering RNA (siRNA), triggered potent and specific gene silencing in mammalian cells (Elbashir et al., 2001a) provided researchers with an unprecedentedly powerful gene silencing tool. The obvious therapeutic potential of siRNAs immediately renewed the scientific and commercial interest in developing nucleic acid drugs capable of low-dose, non-toxic targeting of mRNAs to treat human diseases. As compared to other nucleic acid-based technologies, siRNA benefits from harnessing endogenous RNAi pathways to effectuate gene silencing (Figure 1); upon introduction of synthetic siRNAs into the cell cytoplasm they are incorporated into an RNA-induced silencing complex (RISC; Hammond et al., 2001) by a RISC loading complex (RLC; Maniataki and Mourelatos, 2005) containing the RNase III enzyme Dicer (Bernstein et al., 2001). By sensing the thermodynamic asymmetry of siRNA duplex ends (Khvorova et al., 2003; Schwarz et al., 2003), RLC loads the siRNA guiding antisense strand into a cleavage-competent RISC containing Argonaute 2 (Ago2; Martinez and Tuschl, 2004), whereas the passenger sense strand (SS) is cleaved and released (Matranga et al., 2005; Leuschner et al., 2006). Subsequently, Ago2-RISC will efficiently guide and effectuate multiple rounds of target RNA cleavage resulting in gene “knockdown” (KD; Hutvagner and Zamore, 2002). Furthermore, the structural similarity of endogenous microRNAs (miRNAs) and artificial siRNA triggers may be expected to render these undetectable to cellular sensors of (foreign) dsRNA thereby preventing induction of innate immune-responses. In effect, harnessing siRNA to effectively enter the endogenous RNAi pathway translates into high silencing efficiencies, predictability, and reliability (Bertrand et al., 2002) but concurrently hold the potential to disturb endogenous gene regulation by the native inhabitants of the RNAi pathway, the miRNAs.

## siRNA as a Therapeutic Platform

Small interfering RNAs have gained increasing popularity *in vivo* (Behlke, 2006, 2008; Higuchi et al., 2010; Lares et al., 2010) and the number of RNAi-based preclinical and clinical trials have increased rapidly over recent years with ∼22 different siRNA or short hairpin RNA (shRNA) therapeutics reaching clinical testing for the treatment of at least 16 diseases (http://maps.google.com/maps/ms?ie=UTF8&source=embed&oe=UTF8&msa=0&msid=117696484602143675789.000476c449bf397da6d6c; DeVincenzo et al., 2008; Davis et al., 2010; Leachman et al., 2010; Burnett et al., 2011; Davidson and McCray, 2011; Burnett and Rossi, 2012). This is truly an amazing achievement for a such a fledgling technology considering that conventional development of small-molecule drugs takes at least 5–7 years for preparing a single drug candidate for human clinical trials (Krieg, 2011). For comparison, the first clinical trial of a siRNA-based drug was initiated in 2004 only 3 years after the initial application of siRNA in mammalian cell cultures (Shukla et al., 2010) and successful siRNA designs may easily be adaptable to other target.

### siRNA need chemical engineering to succeed as therapeutic platform

Building superior siRNAs is a combination of choosing an optimal siRNA-target sequence, optimal type of siRNA design, and, importantly, to introduce the proper combination of chemical modifications to suit the particular application. As a scientific tool in mammalian cell cultures, the potency and specificity of unmodified synthetic siRNA may be considered sufficient, yet chemical engineering of siRNAs is a prerequisite to transform them into a novel class of safe therapeutics, a natural progression similar to the development of second generation ASO (Potera, 2007). Recent concerns regarding siRNA delivery and safety have dampened initial excitement and Big Pharma have recently down sized their investments in RNAi R&D (Ledford, 2010; Krieg, 2011; Schmidt, 2011). In particular, the size, lability, and negative charge of siRNAs severely complicate efficient intracellular delivery *in vivo* (Meade and Dowdy, 2009) and siRNA may trigger innate immune-response and lead to unintended deregulation of endogenous gene expression in several ways, as described in the following sections. Encouragingly, these concerns may be fully addressable by careful chemical modification of the synthetic siRNA molecule, an ongoing task that have already gone a long way; the first KD of an endogenous gene, apolipoprotein B (ApoB), was observed in mouse livers after low-pressure intravenous injections of a chemically modified, but naked (non-formulated) siRNA already in 2004 (Soutschek et al., 2004). Also, the first successful KD via RNAi of a cancer target gene in a human, the M2 subunit of ribonucleotide reductase (RRM2), was achieved in 2010 upon systemic siRNA delivery, a holy grail in siRNA therapeutics, using siRNA nanoparticles in a clinical phase-I trial in tumors from melanoma patients (Davis et al., 2010).

## Structural siRNA Designs

**Figure 2:**
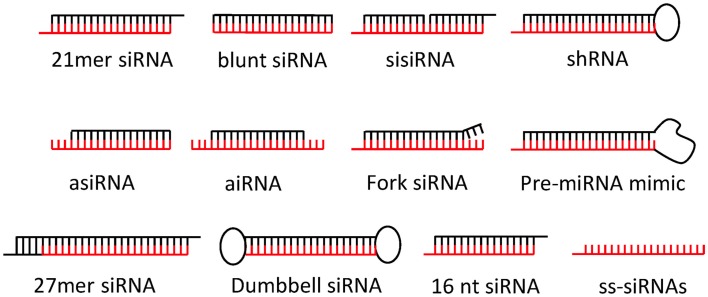
**Popular siRNA design types**. The canonical 21-mer siRNA is the most popular siRNA design today. Dicer-substrate siRNAs such as 27-mer siRNA, shRNA, pre-miRNA mimics, or fork siRNA may enhance siRNA potencies. Asymmetrical siRNAs (aiRNA), asymmetric shorter-duplex siRNA (asiRNA), bulge-siRNAs and sisiRNA may enhance silencing specificity. Blunt-end siRNA are reported to be more nuclease resistant. Single-stranded siRNAs (ss-siRNAs) and 16 mer are functional but may required higher siRNA concentrations. Dumbbell-shaped circular siRNAs may have longer silencing duration. Passenger strands are shown in black and guide strands in red. Please refer to main text for more detail.

Today, a variety of siRNA design types are available for gene silencing each offering benefits and disadvantages (Figure 2): The by far most popular siRNA design mimics natural Dicer cleavage products and comprises a 21 nucleotide (nt) guiding strand antisense to a given RNA target and a complementary passenger strand annealed to form a siRNA duplex with a 19-bp dsRNA stem and 2 nt 3′ overhangs at both ends (here referred to as canonical 21-mer siRNAs; Elbashir et al., 2001a,b). Longer design types, collectively referred to as Dicer-substrate siRNAs (DsiRNAs) structurally mimic various Dicer substrates to enhance incorporation into RNAi pathways and potentially siRNA potency (Kim et al., 2005; Rose et al., 2005; Siolas et al., 2005; Amarzguioui et al., 2006; Collingwood et al., 2008; Hefner et al., 2008; Tanudji et al., 2009). Also shorter or truncated siRNA designs are gaining popularity such as 16-mer siRNA (Chu and Rana, 2008), shRNAs with RNA stems ≤19 bp (Ge et al., 2009a,b), blunt 19-bp siRNAs (Czauderna et al., 2003; Prakash et al., 2005; Hogrefe et al., 2006; Ghosh et al., 2009), asymmetrical siRNAs (aiRNA; Sun et al., 2008), and asymmetric shorter-duplex siRNA (asiRNA; Chang et al., 2009). Finally, fork siRNAs (Hohjoh, 2004; Petrova Kruglova et al., 2010), single-stranded siRNAs (ss-siRNAs; Martinez et al., 2002; Holen et al., 2003; Hall et al., 2006), Dumbbell-shaped circular siRNAs (Abe et al., 2011), bulge-siRNA (Dua et al., 2011), and sisiRNAs (Bramsen et al., 2007) have been successfully utilized, but may require more testing to qualify as a therapeutic siRNA platform. Recently, siRNAs have also been incorporated in larger nucleic acid structures (Afonin et al., 2011; Grabow et al., 2011) with the prospect of enhancing delivery and bio-availability *in vivo*.

## Tolerances for Chemical Modification of siRNAs

The chemical synthesis of siRNAs allows the position-specific incorporation of chemically modified nucleotides in the siRNA to modulate, e.g., thermostability, nuclease resistance, duplex structure, and base-paring properties. For a decade, the compatibility of a diversity of chemical modifications with siRNA function has been mapped by empirical testing. Early siRNA chemical modification schemes quite naturally focused on modification types previously used to potentiate and stabilize ASOs (reviewed in Kurreck, 2003; Wilson and Keefe, 2006) with hopes of similar improvements in siRNA performance. The toolbox of chemical modification types seems ever expanding and current efforts should determine how and which chemical modifications types are best combined in single siRNAs to simultaneously reduce siRNA immunogenicity (Sledz et al., 2003), miRNA-like off-targeting (Jackson et al., 2003), to enhance nuclease resistance/bio-availability *in vivo* (Soutschek et al., 2004; Gao et al., 2009; Merkel et al., 2009), and silencing duration while preserving siRNA potency.

Although effects are naturally rather chemistry-specific, the positional tolerance for chemical modification of siRNAis fairly established (Elbashir et al., 2001b; Chiu and Rana, 2002, 2003; Hamada et al., 2002; Amarzguioui et al., 2003; Braasch et al., 2003; Czauderna et al., 2003; Grunweller et al., 2003; Harborth et al., 2003; Prakash et al., 2005; Choung et al., 2006; Bramsen et al., 2009). As a general trend, the entire passenger strand as well as the 3′-proximal part and 3′ overhang of the guiding stand is most tolerant to chemical modification, which agrees with the observation that only position 2–16 of the guide strand base pairs with its target prior to cleavage (Wang et al., 2009b). The siRNA 3′ overhang of the guide strand, which is bound by the Ago PAZ domain during loading, is conveniently tolerant to chemical modification. This reflects a limited role of PAZ binding during target cleavage (Ma et al., 2004) where the 3′ overhang is released from the PAZ domain (Tomari and Zamore, 2005; Wang et al., 2009b). This renders siRNA 3′ overhangs relatively safe to modify, even with bulky modifications incompatible with the size of the PAZ binding pocket.

In contrast, the 5′ phosphate, the 5′-proximal part, and central positions of the guide strand are sensitive, especially to multiple, thermo-modulating, or bulky modifications that influence the properties of minor groove. These tolerances agree nicely with the structure of the Ago2-guide strand complexes; Here the 5′ phosphate of the guide strand is consistently found in the Ago MID domain (Wang et al., 2008), an essential interaction for strand loading into RISC (Nykanen et al., 2001; Lima et al., 2009). Once bound by Ago2, the initial interactions between the guide strand and target RNA is mediated only by the 5′ proximal siRNA seed region (positions 2–8 of the RISC-associated strand) selectively exposed to the solvent (Wang et al., 2008) and subsequent Ago2-mediated cleavage of target RNA requires forming of an RNA-like A-type helix structure between the guide strand and the target spanning both the seed region and around the cleavage site (opposite of guide strand position 10/11; Meister et al., 2004) hereby explaining the sensitivity of these AS positions to modification.

## Tools for Chemical Modification of siRNAs

**Figure 3:**
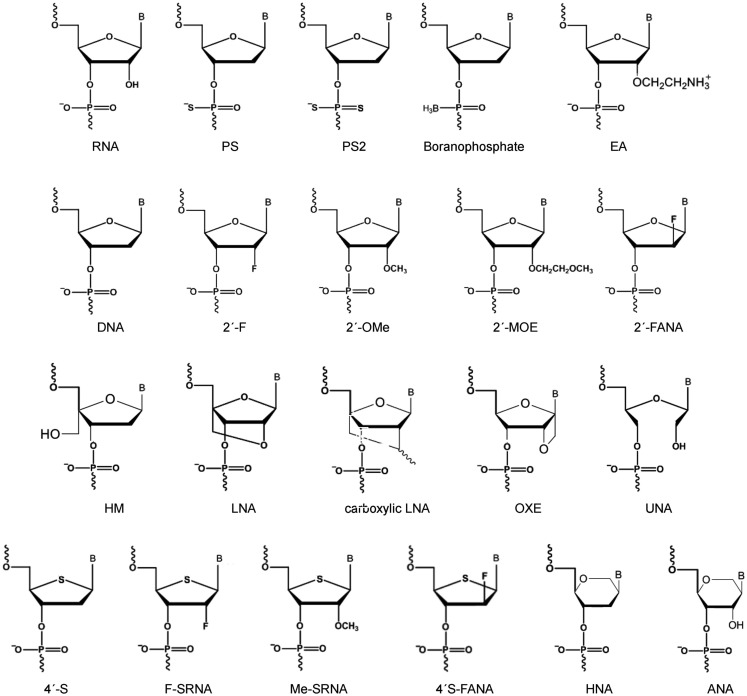
**Popular chemical modification types in siRNA design**. RNA, ribonucleic acid; PS, phosphothioate; PS2, phosphodithioate; EA, 2′-aminoethyl; DNA, deoxyribonucleic acid; 2′-F, 2′-fluoro; 2′-OMe 2′-*O*-methyl; 2′-MOE, 2′-*O*-methoxyethyl; F-ANA, 2′-deoxy-2′-fluoro-β-d-arabinonucleic acid; HM, 4′-*C*-hydroxymethyl-DNA; LNA, locked nucleic acid; carboxylic LNA, 2′,4′-carbocyclic-LNA-locked nucleic acid; OXE, oxetane-LNA; UNA, unlocked nucleic acid; 4′-S, 4′-thioribonucleis acid; F-SRNA, 2′-deoxy-2′-fluoro-4′-thioribonucleic acid; ME-SRNA, 2′-*O*-Me-4′-thioribonucleic acid; 4′-S-F-ANA, 2′-fluoro-4′-thioarabinonucleic acid; ANA, altritol nucleic acid; HNA, hexitol nucleic acid; B, base.

Mainly four classes of chemical modifications is utilized to modify ASOs and now siRNAs: (i) modification of the negatively charged phosphodiester backbone is primarily utilized to enhance siRNA nuclease resistance or affect RNA biodistribution and cellular uptake; (ii) modifications at the ribose 2′-OH group is widely used to modulate most aspects of siRNA behavior including modulating siRNA nuclease resistance, potency, specificity of silencing and to reduce siRNA immunogenicity; (iii) modifications of the ribose ring and nucleoside base is utilized to modulate siRNA stability and base-pairing properties; (iv) dual modifications harbor two modified functionalities in a single nucleotide and especially the combination of backbone and ribose modifications with 2′ OH substitutions are currently gaining momentum (for a non-exhaustive selection of popular chemical modification types in siRNA design see Figure 3).

### siRNA backbone modification

A classic and popular phosphate backbone alteration is the phosphoromonothioate (PS) modification where one of the non-bridging phosphate oxygens is replaced with sulfur (Braasch et al., 2004). Also phosphorodithioate (PS2) substitutions, where both non-bridging oxygen atoms are replaced, were recently tested in siRNA designs and slightly increased siRNA potencies and nuclease resistance as compared to PS and unmodified siRNA (Yang et al., 2012). Moderately PS-modified siRNAs support efficient RNAi, yet effects are very position-dependent (Amarzguioui et al., 2003; Braasch et al., 2003; Chiu and Rana, 2003; Grunweller et al., 2003; Harborth et al., 2003; Choung et al., 2006) but extensive PS modification reduces silencing (Chiu and Rana, 2003; Hall et al., 2004) and has toxic side-effects (likely due to a tendency to bind non-specifically to cellular membrane proteins; Amarzguioui et al., 2003; Harborth et al., 2003). Still, moderate PS modification levels have been widely and successfully combined with ribose 2′-OH modifications in both ASO and siRNA designs to stabilize naked RNA for systemic application in mice (Soutschek et al., 2004; Elmen et al., 2008). Both PS and PS2 will slightly thermo-destabilize siRNA duplexes (∼0.5°C per modification; Eckstein, 2002; Amarzguioui et al., 2003; Harborth et al., 2003; Yang et al., 2012). Substitution of the phosphodiester linkage with a boranophosphate linkage have been explored in gene silencing using a canonical siRNA (Hall et al., 2004) or single-stranded siRNAs (Hall et al., 2006). Similarly to PS and PS2, boranophosphate linkages decrease the *T*_m_ of RNA duplexes by ∼0.5–0.8°C per modification (Li et al., 2007) and enhances nuclease resistance as compared to unmodified siRNAs, yet is better-tolerated in siRNA design than PS (Hall et al., 2004). Still this modification type has not been widely used.

A phosphonoacetate (PACE) modification consists of an acetate group in place of a non-bridging oxygen in an internucleotide phosphate linkage whereas a thioPACE modification is an analogous substitution in a phosphorothioate linkage. Both modifications are completely resistant to degradation and PACE is electrochemically neutral if esterified with, e.g., methyl groups (Sheehan et al., 2003), which allows modified oligonucleotides to be taken up by cells in the absence of delivery reagents (Yamada et al., 2007). Also dual modification types combining 2′-OMe and PACT/ThioPACT have recently been tested in siRNA design (see below). Finally, amide linkages (Iwase et al., 2007) and 2′,5′-linkages (Prakash et al., 2006) have been found to enhance nuclease resistance of siRNAs, yet are little used so far.

### Ribose 2′-OH substitutions

Modifications of the ribose 2′-OH is the most popular approach in siRNA design in which the 2′-OH is either substituted with other chemical groups or the 2′-oxygen is “locked” via intramolecular linkages as in bridged nucleic acids (BNAs). So far, the small electronegative 2′ substituents such as 2′-fluoro (2′-F), DNA (2′-H), 2′-*O*-methyl (2′-OMe) are most widely used as they are well-tolerated, will generally enhance siRNA nuclease resistance and not dramatically affect siRNA thermostability, dsRNA duplex conformation nor siRNA activity.

Fluorine substitution (2′-F) slightly stabilizes dsRNA duplexes (∼1°C increase in *T*_m_ per modification; Freier and Altmann, 1997; Allerson et al., 2005), is among the best tolerated modification types and therefore allows the creation of highly modified, active siRNAs: both strands tolerate 2′-F modification at most positions (Braasch et al., 2003; Chiu and Rana, 2003; Harborth et al., 2003; Prakash et al., 2005; Choung et al., 2006; Manoharan et al., 2011) and substitution of all siRNA pyrimidines was reported to greatly enhance serum stability and to support effective silencing *in vitro* and *in vivo* (Capodici et al., 2002; Layzer et al., 2004; Morrissey et al., 2005a). Also, 2′-F has proven superior during an application *in vivo* using a mouse model for silencing of the factor VII gene as directly compared to other 2′ OH modifications types such as the popular 2′-OMe (Manoharan et al., 2011). Even fully substituted siRNA containing alternating modifications of 2′-F and DNA (Blidner et al., 2007) or 2′-OMe (Allerson et al., 2005) can be both highly potent and nuclease resistant.

Similarly to 2′-F, the naturally occurring 2′-OMe modification was among the first and most extensively tested 2′-substitutions (Amarzguioui et al., 2003; Chiu and Rana, 2003; Czauderna et al., 2003; Grunweller et al., 2003; Prakash et al., 2005; Choung et al., 2006; Jackson et al., 2006a); 2′-OMe slightly enhances the binding affinity toward RNA (*T*_m_ increase of 0.5–0.7°C per modification) and is well-tolerated in most duplex positions (Freier and Altmann, 1997; Kraynack and Baker, 2006), although less so than 2′-F in the guide strand. More extensive or full modification, particularly of the guide strand seed region, can reduce siRNA potency (Elbashir et al., 2001a; Braasch et al., 2003; Chiu and Rana, 2003; Czauderna et al., 2003) although conflicting results suggest that tolerances are siRNA sequence-dependent (Choung et al., 2006; Kraynack and Baker, 2006). Traditionally, 2′-OMe modification has been extensively used to increase siRNA nuclease resistance and proven particular successful in combination with other 2′-modifications, e.g., 2′-F, to generate fully substituted, nuclease resistant, yet functional siRNAs (Allerson et al., 2005). Also, partially 2′-OMe/PS-modified siRNA conjugated with cholesterol was the first chemical design to successfully silence an endogenous gene in mice using a systemic delivery strategy suitable for therapeutics (Soutschek et al., 2004). More recently 2′-OMe modification is also gaining popularity for reducing siRNA immunogenicity and off-targeting (see Reducing siRNA Immunogenicity by Chemical Modification and Reducing siRNA Off-Target Effects by Chemical Modification).

DNA modification, typically dTdT, has long been the industry standard for modifying siRNA 3′ overhangs to reduce cost, while conferring nuclease resistance (Elbashir et al., 2001a) and preserving siRNA potency (Braasch et al., 2003; Chiu and Rana, 2003). As for 2′-F and 2′-OMe, DNA is well-tolerated in the passenger strand with little effect on siRNA potency (Hogrefe et al., 2006; Pirollo et al., 2007). Similarly to 2′-OMe, only partially DNA substituted guide strands are functional (Parrish et al., 2000; Chiu and Rana, 2003) whereas alternating the DNA modification with 2′-F has created fully substituted, active guide strands (Chiu and Rana, 2003). Notably, full DNA substitution of the guide strand seed region can reduce off-target effects (see Reducing siRNA Off-Target Effects by Chemical Modification) albeit its influence on siRNA potencies may be somewhat sequence-dependent (Ui-Tei et al., 2008) and dTdT overhangs seem to reduce silencing duration (see Strapps et al., 2010; Enhancing Silencing Duration).

More bulky 2′-modifications such as 2′-*O*-MOE, 2′-*O*-allyl and others are only tolerated at certain positions within the siRNA duplex, likely owing to their distortion of RNA helix structure essential to Ago2 cleavage (Amarzguioui et al., 2003; Prakash et al., 2005; Odadzic et al., 2008; Bramsen et al., 2009). This somewhat limits their use for general siRNA design as similar enhancements in, e.g., nuclease resistance may be obtained by the better-tolerated, smaller 2′ substitutions described above. Still, bulky 2′-modifications are best tolerated at the 3′-ends of siRNA strands (Odadzic et al., 2008), where they may be used to modulate thermodynamic stability and duplex asymmetry to enhance siRNA potency: e.g., an 2′-aminoethyl modification in the passenger strand 3′-end can enhance siRNA potency, likely by affecting strand selection during RISC loading (Bramsen et al., 2009). Still, bulky 2′-modifications are not widely used other than in siRNA overhangs to enhance nuclease resistance (Amarzguioui et al., 2003; Prakash et al., 2005; Odadzic et al., 2008; Bramsen et al., 2009).

### Bridged nucleic acids

Another class of 2′-modification is the BNA in which the ribose 2′-oxygen is linked to the 4′-carbon via a methylene bridge as in Locked Nucleic Acid (LNA; Wengel et al., 2001) and carbocyclic-LNA (Srivastava et al., 2007; Bramsen et al., 2009), via an ethylene bridge as in ethylene-bridged nucleic acid (ENA; Hamada et al., 2002) and carbocyclic-ENA (Srivastava et al., 2007; Bramsen et al., 2009), or to the 1′-carbon as in oxetane (OXE; Pradeepkumar et al., 2003; Bramsen et al., 2009). This radical modification type generates nucleotides with interesting properties to siRNA design; The methylene bridge in the very popular LNA-modification locks the sugar moiety in the RNA-helical C3′-*endo* conformation, which additively increase RNA duplex thermostability by 2–10°C per LNA (Petersen and Wengel, 2003). Although this dramatic thermo-stabilization limits siRNA modification levels by LNA (Braasch et al., 2003; Grunweller et al., 2003; Elmén et al., 2005), it provides unique opportunities to modulate the local thermodynamic profile within the siRNA duplex to optimize strand selection and thereby enhance specificity (Elmén et al., 2005; Bramsen et al., 2007, 2009), to enhance nuclease resistance *in vitro* (Braasch et al., 2003; Bramsen et al., 2009) and *in vivo* (Mook et al., 2007; Glud et al., 2009) and reduce siRNA immunogenicity (Hornung et al., 2005) as described in the following sections. Also, the thermo-stabilizing LNA may be used to ensure the integrity of siRNA designs relying on only short regions of oligo base-pairing such as the three stranded sisiRNA design (Bramsen et al., 2007). As a note of caution, LNA-modified ASO scan induce profound hepatotoxicity in mice (Swayze et al., 2007) albeit this is not observed in other studies, e.g., in primates (Elmen et al., 2008).

### Alteration of the ribose moiety

Modification types based on sugar moieties other than ribose have been successfully used in siRNA designs upon incorporation of, e.g., altritol nucleic acid (ANA), hexitol nucleic acid (HNA), 2′-deoxy-2′-fluoroarabinonucleic acids (2′-F-ANA), and cyclohexenyl nucleic acid (CeNA) nucleotides, which are based on altritol, hexitol, arabinose, and cyclohexenyl sugars, respectively (Dowler et al., 2006; Fisher et al., 2007, 2009; Nauwelaerts et al., 2007; Watts et al., 2007; Bramsen et al., 2009).

The 2′-F-ANA modification is structurally similar to DNA (C2′-*endo* conformation) and increases the *T*_m_ of the siRNA duplex by ∼0.5–0.8°C per modification and will, due its structural similarity to DNA, structurally distort siRNA duplexes making it little suited to modify the seed regions of the guide strand. Full modification of the SS can lead to significant enhancements in potency and nuclease resistance and 2′-F-ANA-modification may be particular useful in the guide strand to create high-affinity 3′ overhangs, similarly to DNA (Dowler et al., 2006; Watts et al., 2007). Moreover, heavily modified siRNAs that contain combinations of 2′-F-ANA and 2′-F or LNA show superior properties (Deleavey et al., 2010).

The ANA modification will slightly enhance siRNA thermostability, are stable against enzymatic degradation and can moderately enhance siRNA activity and silencing duration upon incorporation into of both duplex 3′-ends (Fisher et al., 2007) whereas overhang modification slightly decrease RISC affinity (Maiti et al., 2011). Similarly, the incorporation of HNA at both strand 3′-ends enhanced silencing potency, serum stability, and silencing duration of a siRNA against B-Raf, even more so than observed for ANA-modifications upon direct comparison (Fisher et al., 2009). Finally, CeNA modification of passenger and guide stand 3′-ends can increase siRNA potency and nuclease resistance (Nauwelaerts et al., 2007).

A radical modification of the ribose is found in unlocked nucleic acid (UNA) which is an acyclic derivative of RNA lacking the C2′–C3′-bond of the ribose ring that still structurally mimic unmodified RNA upon incorporation into RNA duplexes. Rather uniquely, the incorporation of UNA residues strongly destabilizes the duplex by 5–8°C per UNA monomer hereby allowing dramatic local destabilization of siRNA duplex and guide strand/target interactions (Langkjær et al., 2009). As a consequence, extensive UNA modification will prevent annealing of siRNA strands and limits modification levels to 2–3 UNAs within the duplex region (Laursen et al., 2010). Still, even modest UNA modification has proven very successful in enhancing siRNA biostability and function *in vivo* (Laursen et al., 2010), in preserving the potency of extensively thermo-stabilized siRNA (such as in extensively LNA-modified siRNAs; Laursen et al., 2010) and in reducing siRNA off-targeting (Bramsen et al., 2010; Vaish et al., 2011) as described in Section “Reducing siRNA Off-Target Effects by Chemical Modification.”

Finally, 4′-thio-modified nucleotides (4′-S RNA) contain a sulfur atom instead of the 4′-carbon of the ribose ring and can enhance nuclease resistance, target affinity, and siRNA potency. Stretches of 4′-thio-RNA were well-tolerated in both siRNA strands albeit only at certain positions in the guide strand (Hoshika et al., 2005; Dande et al., 2006) and sequence-specific effects of 4′-thio modifications have been reported (Hoshika et al., 2007). Instead, 4′-thio-RNA can be utilized as a “dual modification” by combining it with 2′-OMe (Dande et al., 2006) or 2′-F (Takahashi et al., 2009), as described below.

### Base modification

Chemical modification of the RNA nucleobases is still less commonly used in siRNA design (reviewed in Peacock et al., 2011). A number of modified nucleotide bases, such as 5-bromo-, 5-iodo-, 2-thio-, 4-thio-, dihydro-, and pseudo-uracil, have been tested with the prospect of to stabilize base-pairing and to enhance base paring specificity; 5-bromo- and 5-iodo-uracil slightly reduced siRNA potency (Chiu and Rana, 2003), whereas 2-thio- and pseudo-uracil were reported to enhance siRNA potency (Sipa et al., 2007) and to reduce cellular immune responses (Hornung et al., 2006).

### Dual Modifications

The combination of two modified functionalities on a single ribose in so-called “dual modification” types has recently been tested in siRNA design to address that 2′-modification or backbone modification alone typically confers insufficient nuclease resistance and pharmacokinetic properties for siRNA applications *in vivo*.

The 4′-Thio (4′-S) modification has been combined with a variety of 2′-substitutions such as 2′-OMe, 2′-*O*-MOE, 2′-F, or 2′-F-ANA (Dande et al., 2006; Watts et al., 2007; Takahashi et al., 2009); Most significantly, 2′-OMe/4′-S modification can significantly enhance serum stability as compared to 2′-OMe (or 2′-F) modification alone and does not affect duplex thermostability, whereas 2′-F-ANA/4′-S increase the *T*_m_ by ∼1°C per modification but does not improve serum stability beyond simple 2′-OMe modification (Takahashi et al., 2009). Recently, 2′-OMe/4′-S modification was shown to significantly enhance siRNA silencing longevity, an effect that was attributed to enhanced intracellular siRNA stability (Takahashi et al., 2012). The 2′-F-ANA/4′-S modification, which decreases the *T*_m_ ∼ 1.0–1.4°C per modification, is only tolerated at low modification levels and does not immediately offer unique advantages in siRNA potencies (Watts et al., 2007).

Combining 2′-OMe and PS modification to create 2′-OMePS has generated fully modified passenger strands with dramatically enhanced nuclease resistance whereas similar modification rendered guide strands inactive (Kraynack and Baker, 2006). This remarkable nuclease resistance seems well suited for ASO design: in a phase-I clinical trial, the 2′-OMePS-modified ASO, PRO051, could successfully restore specific exon skipping during mRNA splicing leading to restoration of dystrophin expression in patients with Duchenne’s muscular dystrophy (van Deutekom et al., 2007).

Combination of 2′-OMe and PACT or thioPACT linkages decrease the *T*_m_ of RNA duplexes ∼0.75–1°C per modification as compared to 2′-OMe-modified duplexes alone (which are stabilized compared to unmodified dsRNA; see above; Threlfall et al., 2012). PACT and ThioPACT enhance siRNA stability, and will reduce siRNA potency, even if only placed in the passenger strand, and (double-stranded) siRNA are not efficiently taken up by cells in the absence of transfection reagents, in contrast to modified single-stranded oligonucleotides (Threlfall et al., 2012). Finally, 4′-aminomethyl-2′-OMe dual modification of siRNAs will slightly lower the *T*_m_ (∼1°C/modification) compared to the unmodified duplex, can increase siRNA serum stability and modifications are well-tolerated at various positions in the passenger strand, but not in the seed region of the guide strand (Gore et al., 2012).

## Designing Potent siRNAs by Chemical Engineering

A natural priority in siRNA design is to ensure maximal gene silencing at low concentrations, i.e., to obtain IC_50_ values in the nano or pico-molar range suitable for silencing *in vivo*, where siRNA delivery is often difficult. Important knowledge of the most influential factors for siRNA potency has been obtained by empirical testing of large libraries of unmodified siRNAs in cell culture and subsequent bio-informatical data mining (Reynolds et al., 2004; Huesken et al., 2005; Vert et al., 2006; Ladunga, 2007; Klingelhoefer et al., 2009; Wang et al., 2009a). Several studies propose nucleotide base preferences at selected positions relative to the target sequence such as G/C at position 1, A/U at positions 10 and 19, more than three A/Us between positions 13 and 19 (Amarzguioui and Prydz, 2004; Reynolds et al., 2004; Ui-Tei et al., 2004; Jagla et al., 2005), and position-independent overrepresentation of certain di- and tri-nucleotide motifs (Ebalunode et al., 2011), and relatively U-rich antisense strands (Ladunga, 2007; Wang et al., 2010), especially in the 5′-end (Ui-Tei et al., 2004). An obvious question is whether chemical modification can enhance the potencies of already well-designed unmodified siRNAs. Indeed, numerous studies report the improvement of siRNA potency by chemical modification; typically improvements in potency are moderate, less than twofold, and only in few cases has chemical modification led to dramatic enhancements of siRNA potency (Allerson et al., 2005), effects, which may be too sequence-specific for general siRNA design (Koller et al., 2006). Hereby chemical modification strategies may not necessarily create “super-potent” siRNAs (as compared to well-designed unmodified siRNAs), but can be efficiently used to counteract reductions in potency encountered while addressing other siRNA shortcoming in siRNA stability, off-targeting, immunogenicity, or to tweak siRNA thermodynamics when targeting a defined target with a non-optimal sequence composition.

### Modulating siRNA thermostability and -asymmetry to enhance siRNA potency

Of interest to siRNA chemical modification strategies it is notable that most of the siRNA sequence preferences described above may reflect an optimal siRNA thermodynamic profile that ensures efficient handling by RNAi proteins. Most prominently, siRNA strand selection during RISC activation is determined by the thermodynamic asymmetry between siRNA duplex ends: the siRNA strand having its 5′-end embedded at the thermodynamically least stable end is preferentially employed as guide stand within RISC (Khvorova et al., 2003; Schwarz et al., 2003). Consequently, siRNAs should be designed to be thermodynamically asymmetric either by simply choosing AU base pairs at the 5′ terminal positions of the guide stand and/or GC base pairs in its 3′-end or, more advanced, by engineering proposed optimal difference in Gibbs free energy between duplex ends (Khvorova et al., 2003; Shabalina et al., 2006), either by sequence choice or by introducing mismatches or chemical modifications. In this regard, many chemical modification types are ideal tools to position-specifically modulate siRNA thermostability; modifications such as LNA, 2′-F, 2′-OMe are stabilizing, whereas other such as UNA, OXE, and PS are destabilizing. Chemical screens have identified that modifications ensuring preferential loading of the intended guide strand into RISC will often enhance potency; destabilizing modification in the passenger duplex 3′-end such as OXE, ethylamino, UNA, dihydrouracil, or PS (Elmén et al., 2005; Li et al., 2005; Bramsen et al., 2009; Yang et al., 2012) and stabilizing modification in the passenger strand 5′-end such as LNA (Elmén et al., 2005) and/or stabilizing modification in the guide strand duplex 3′-end improve siRNA potency (Bramsen et al., 2009). For this purpose, highly stabilizing or destabilizing chemical modifications are not frequently used in the guide strand seed region as they can dramatically reduce siRNA efficiency (Bramsen et al., 2009). It is indeed likely that most of the modification schemes proposed to moderately enhance siRNA potencies by modifying strand 3′-ends is indeed ensuring optimal thermodynamic asymmetry.

The thermo-stability profile of the internal part of the siRNA duplex seem also important as it influences rates of siRNA duplex unwinding during RISC loading and target RNA release; Design of a relatively loose siRNA duplex with an overall GC-content of 30–50% has been common practice (Wang et al., 2009a), yet more recently Gibbs free energy calculations by the nearest neighbor method has predicted efficient siRNAs and an energy cut-off (Δ*G* < −34.6 kcal/mol) was introduced to reject too thermo-stable siRNAs (Ichihara et al., 2007). It remains, however, to be fully established if the preference for GC-poor siRNAs may rather indirectly reflect a higher accessibility of AU-rich target sites within the structured mRNA; in fact, target site accessibility is suggested to be more important to siRNA efficiency than GC-content *per se* (Chan et al., 2009).

A few studies have devised how the activity of too thermo-stable and therefore little potent siRNA, typically seen upon extensive LNA, 2′-OMe/2′-F modification, can be rescued by introducing counteracting modifications like destabilizing UNA modification (Laursen et al., 2010) or introducing a nicked passenger strand as in the so-called sisiRNA design (Bramsen et al., 2007).

### Enhancing siRNA potency by siRNA overhang modification

Preferential guide strand loading into RISC can also be helped by modifying the canonical siRNA 2 nt 3′ overhangs, which are bound by the PAZ domain Ago during RISC loading (Ma et al., 2004). The PAZ domain was originally described to bind siRNA overhangs in a sequence unspecific manner (Lingel et al., 2004; Ma et al., 2004), yet siRNA overhang composition does influence their affinity for the Ago2 PAZ domain and siRNA potencies: particularly the UU overhang has high affinity for the PAZ domain but is concurrently easily dissociated from the PAZ domain upon strand cleavage (Lee et al., 2007). In agreement, superior silencing of siRNA with UU overhang as compared to AA, GG, and CC has been reported (Elbashir et al., 2001b). Hereby, differential use of UU in the guiding strand and GG, CC pairs in the passenger strand should ensure preferential loading of the guide strand into RISC. Also, the siRNA overhangs can, due to base stacking, contribute nearly the same to the thermodynamic stability as a regular base pair (O’Toole et al., 2005) and thereby help to direct siRNA strand selection by altering the siRNA thermodynamic asymmetry, a somewhat overlooked strategy. Finally, preferential guide strand loading into RISC can be helped by chemically modifying the siRNA 3′ overhangs hereby providing a sequence-independent, general strategy for enhancing siRNA performance. Modification types such as DNA, LNA, UNA, etc. that favor or disfavor strand selection during RISC loading have been identified and can be used in the guide and passenger strand, respectively, to enhance siRNA potency and specificity (Elbashir et al., 2001b; Dowler et al., 2006; Bramsen et al., 2009).

## Designing Nuclease Resistant siRNAs by Chemical Engineering

The general high susceptibility of single-stranded RNA to ribonuclease degradation (Tsui et al., 2002) and initial observations that unmodified siRNAs are subject to relatively fast serum degradation (Czauderna et al., 2003; Choung et al., 2006) suggested that siRNA would need chemical stabilization to succeed as a systemically applied RNA therapeutic. Indeed, the subsequent observation that chemical stabilization was essential for successful silencing by unformulated siRNAs following intravenous injection in mice only consolidated this point and made chemical stabilization a integral part of siRNA engineering. In essence, however, chemical stabilization is little needed for typical siRNA applications in cell culture where commercial transfection reagents will shield the siRNAs from serum RNases. In a therapeutic setting siRNA may also only need extensive chemical stabilization if exposed to extracellular environments such as the blood stream, digestive tract, lung, or skin, in particular if delivered unformulated, and modification strategies for formulated siRNAs need rather focus on reducing siRNA immunogenicity, off-targeting without jeopardizing silencing potency/duration. Still, chemical stabilization may enhance silencing duration (see Enhancing Silencing Duration) and application of naked, chemically modified siRNA is still a popular approach with both formulated and unformulated siRNAs currently undergoing pre- and clinical investigation (Schmidt, 2011).

### Sequence-specific siRNA degradation by extracellular ribonucleases

Compared to ssRNA, dsRNAs are rather poor substrates for most RNases and siRNA degradation is highly sequence-dependent upon incubation in blood serum, a popular mimic of “extracellular” conditions *in vivo* (Choung et al., 2006; Haupenthal et al., 2006; Hong et al., 2010). Here siRNA degradation is mediated primarily by RNase A-like activities (Haupenthal et al., 2006, 2007; Turner et al., 2007) that, being pyrimidine-specific endonucleases, cleave dsRNA at specific dinucleotide motifs such as UpA, CpA, and UpG (Qiu et al., 1998; Sorrentino, 1998; Turner et al., 2007; Volkov et al., 2009). Recent efforts have characterized siRNA degradation products to identify particularly vulnerable sequence-motif and indeed degradation in serum occur at only few sites, primarily at UA and CA dinucleotides (Choung et al., 2006; Turner et al., 2007; Volkov et al., 2009). It is still not fully clear how the siRNA thermodynamic stability influences endonuclease resistance; seminal RNases are reported to recognize vulnerable single-stranded regions exposed from the duplex by thermal fluctuations predicting that thermo-stable siRNA would be more nuclease resistant. In accordance, thermodynamically “loose” regions within the siRNA duplex, which is deliberately introduced in the siRNA by design to ensure guide strand RISC loading, are reportedly more prone to nuclease attack than thermodynamically more stable regions (Haupenthal et al., 2006). In contrast, a comprehensive study found no general thermodynamic difference between the vulnerable regions and the remaining siRNA and that both strands were cleaved simultaneously (almost exclusively at UA and CA motifs) arguing against recognition of exposed single-stranded motifs (Hong et al., 2010). These discrepancies aside, avoidance or modification of UA and CA motifs within the duplex stem is an attractive strategy to enhance endonuclease resistance.

Notably, blunt-ended siRNA are reportedly more stable in serums than their canonical counterparts (Czauderna et al., 2003) leading to the suggestion that exo-nucleases are dominant mediators of siRNA degradation in serum (Soutschek et al., 2004). However, latter studies report little indications for high exonuclease activity in serum (Haupenthal et al., 2006; Turner et al., 2007) and propose that the siRNA 2 nt 3′ overhangs may reduce siRNA stability in serum by allowing ribo-endonucleases to dock on the siRNAs (Haupenthal et al., 2006). In any case, chemical modification of siRNA overhangs is a popular approach to slightly enhance siRNA nuclease resistance as described below.

### Intracellular siRNA stability

Small interfering RNAs are far more stable once inside the cell and silencing can last 30–90 days in slowly or non-dividing cells (Song et al., 2003; Bartlett and Davis, 2007); It is likely that some stability is gained upon incorporation into RNAi protein complexes such as RISC, however, double-stranded siRNAs are reported to be quite stable intracellular in contrast to their corresponding single strands (Raemdonck et al., 2006). Still, the 3′ exonuclease Eri-1 (Kennedy et al., 2004; Takabatake et al., 2007) has been described as a key mediator of intracellular siRNA degradation and high doses of siRNA can enhance Eri-1 levels suggesting that the RNAi machinery may be controlled by a negative feedback mechanism (Hong et al., 2005; Bian et al., 2011).

### Enhancing siRNA nuclease resistance by chemical modification

The most common route of non-specific degradation, which is also catalyzed by several nucleases, involves a nucleophilic attack of the ribose 2′-OH and hydrolysis of the interphosphate linkage via a 2′,3′-cyclic phosphate intermediate (Usher, 1969). Therefore, siRNA nuclease resistance is enhanced by modifying the ribose 2′OH groups or the internucleotide phosphate linkage. As extensive chemical modification tends to reduce siRNA potencies the challenge is to identify modification schemes that balance stability and silencing potency in respect to the specific delivery strategy, e.g., siRNA formulation and delivery route.

With the assumption that more modifications would generate better serum stability, early efforts to prepare siRNA for naked systemic delivery aimed to maximize the number of modified positions within siRNA duplex. Especially, the combination of moderate phosphoromonothioate (PS) backbone modification with small, well-tolerated 2′ substitutions (2′-F, 2′-OMe, and 2′-H) or even fully modified siRNAs have often created highly stable siRNA for applications *in vivo* (Soutschek et al., 2004; Allerson et al., 2005; Morrissey et al., 2005b; Zimmermann et al., 2006; Blidner et al., 2007; Nishina et al., 2008). In some cases, modification schemes can be very complex; a successful example is a siRNA having a passenger strand with 2′-F substitutions on all pyrimidine positions, DNA in all purine positions, 5′ and 3′-inverted abasic end caps and a guide strand with 2′-F substitutions in all pyrimidine positions, 2′-OMe substitutions of all purines and a single PS modification at the 3′ terminal linkage that produced dose-dependent gene silencing upon intravenous injection in mice (Morrissey et al., 2005b). Extensively modified siRNA is still the norm for systemic administration and future commercial siRNA therapeutics may likely be fully modified siRNA harboring complex modification schemes, at least if not protected by formulation. Yet, for general siRNA design, complicated modification schemes hold the obvious pitfall of dramatically reducing siRNA functionality and siRNA sequence dependencies are not always fully understood. Also, researches may find the task of testing multiple heavily modified siRNAs to identify potent candidates for applications *in vivo* both technically and financially challenging.

The recent pinpointing of siRNA dinucleotides vulnerable to nuclease attack suggest that their selective chemical modification may represent a general strategy to greatly enhance siRNA nuclease resistance while preserving siRNA potency (Turner et al., 2007; Armstrong et al., 2008; Volkov et al., 2009). Typically, the well-tolerated 2′-OMe and 2′-F modifications are used within the duplex stem to modify UA and CA motifs and can dramatically improve nuclease resistance with little impact on siRNA potency (Turner et al., 2007; Armstrong et al., 2008; Volkov et al., 2009; Hong et al., 2010). Also, as siRNA 3′ overhangs are stabilized by most modification types, these may well be chosen to simultaneously ensure preferential loading of the guide strand (see above), e.g., the LNA modification proposed by Bramsen et al. (2009) will simultaneously enhance stability and potency. Notably, UNA-modified overhangs were found to dramatically enhance the serum stability of otherwise unmodified siRNAs in mouse serum leading to successful gene silencing *in vivo*, whereas little difference in stability was seen in bovine serum (Laursen et al., 2010) likely reflecting species differences in RNase composition or serum preparation; As a note of caution, degradation of siRNAs is faster in human than mouse serum suggesting that future therapeutic application of unformulated siRNAs may prove more difficult in humans than in mouse models (Haupenthal et al., 2006).

Alternatively, siRNAs may be stabilized by moderately enhancing their local thermodynamic stability to minimize the accessibility of vulnerable sequence-motifs exposed by thermal fluctuations, e.g., the introduction of stabilizing LNA (Braasch et al., 2003; Grunweller et al., 2003; Elmén et al., 2005; Mook et al., 2007; Bramsen et al., 2009) or 4′ thioribose (Dande et al., 2006) at selected positions within the duplex can enhance siRNA stability while generally preserving siRNA potency. Notably, siRNAs are designed to be thermodynamically asymmetric and hereby more “loose” in one duplex end hereby rendering it particular exposed to endonuclease attack, a problem that has been addressed by 2′-OMe modification of loose regions (Hoerter and Walter, 2007).

## Enhancing Silencing Duration

The transient nature of gene silencing by synthetic siRNA poses concerns for therapeutics as it will require repeated dosing at short intervals. Such transience is primarily due to the continuous dilution of the effective intracellular siRNA pool through cellular proliferation and degradation; in cultures of proliferating cells, silencing duration is most dependent on dilution through cell division and silencing normally persists for no more than 2–7 days whereas this period can be extended for several weeks in terminally differentiated non-dividing cells, such as macrophages, and up to 1 month in the slowly dividing CCD-1074Sk cells (Bartlett and Davis, 2007). Encouragingly, this implies that silencing duration may be much longer in a therapeutic setting where target cells usually are slowly or non-dividing and indeed long-lasting target inhibition in the livers of mice and non-human primates have been reported (Zimmermann et al., 2006).

### Sequence-dependent silencing duration

Small interfering RNA silencing duration appears to be sequence-dependent and does not necessarily correlate with initial silencing efficacy (Strapps et al., 2010). The basis for such sequence-dependency remains unresolved but may reflect multiple factors such as the differences in intracellular stability of unbound siRNA, their affinity for RNAi pathway components, reaction kinetics and target (and off-target) levels (Arvey et al., 2010). Also, endogenous proteins that bind siRNA and dampen silencing activity, such as Eri-1 (Kennedy et al., 2004; Bian et al., 2011) and ADAR1 (Hong et al., 2005), may have sequence-dependent affinities for siRNAs. Notably, the expression of Eri-1 and ADAR1 are induced by high-doses of siRNA and may account for the observation that higher doses of siRNA in some instances had lower silencing duration than lower doses both in cell cultures and in mouse livers upon hydrodynamic injection (Hong et al., 2005; Bian et al., 2011).

### Enhancing siRNA silencing longevity by chemical stabilization

Many studies attribute the prolonged silencing duration of chemically modified siRNAs to their high nuclease resistance which would ensure delivery of more intact siRNAs into the cell and subsequently stabilize the internalized siRNA pool available for RISC recruitment. Several of the stabilizing modifications mentioned above have also been reported to slightly enhance the duration of silencing using 2′-OMe (Collingwood et al., 2008; Volkov et al., 2009), F-ANA (Dowler et al., 2006), 2′-F (Chiu and Rana, 2003), or complex modification schemes (Morrissey et al., 2005b), whereas other studies do not find enhancement of silencing persistence upon chemical stabilization (Layzer et al., 2004). A direct comparison of stabilized and unmodified siRNAs delivered by several different methods *in vitro* and *in vivo* have suggested that the benefits of chemical stabilization arise during siRNA delivery prior to cellular internalization and that stabilization does not significantly enhance silencing magnitude or persistence once siRNA are located in the cytoplasm (Bartlett and Davis, 2007). Still, Takahashi et al. (2012) reported that the higher intracellular stability of a 2′-OMe-4′-Thio-modified siRNA, as evaluated by QPCR, leads to long-term RNAi effect suggesting that previous modification schemes may not have provided sufficient stability to significantly enhance silencing persistence. In agreement, Volkov et al. (2009) found that prolonged silencing could only be identified in cell culture when all vulnerable dinucleotide motif were 2′-OMe-modified within the siRNA.

### siRNA recruitment and silencing duration

Notably, the industry-standard DNA overhangs, typically dTdT, has been reported to significantly reduce silencing duration irrespectively of the siRNA sequence (Strapps et al., 2010) and it is tempting to speculate that the reported sixfold higher affinity of dTdT-siRNA for Ago2 (Maiti et al., 2011) may in fact reduce silencing duration as siRNAs are faster recruited and turned over than lower affinity siRNAs. In accordance with this, rates for siRNA-RISC complex formation is two times slower for blunt siRNAs compared to canonical siRNA, whereas silencing persistence is enhanced (Ghosh et al., 2009). Also, the dumbbell siRNA design (Abe et al., 2007), which encompasses a 23-bp stem and 9-nt loops at both ends, exhibits a prolonged silencing persistence potentially because Dicer only very slowly processes RNA dumbbells into siRNA species (Abe et al., 2011).

## Reducing siRNA Immunogenicity by Chemical Modification

The original observation that synthetic 21-mer siRNA can trigger sequence-specific mRNA degradation without apparent side-effects in human cell cultures promised synthetic siRNA to bedrock efficient and safe RNA therapeutics (Elbashir et al., 2001a). This observation was especially appreciated as the introduction of long dsRNA, a gene silencing strategy employed in popular non-mammalian systems, had been precluded in mammals by the induction of innate immune responses leading to pro-inflammatory cytokine production and ultimately cell death (Minks et al., 1979). It is becoming increasingly clear, however, that unmodified synthetic siRNA can be triggers of innate immune responses depending on their structure, sequence, target cell type, entry route, formulation reagent used and concentration, despite their structural mimicry of endogenous siRNA/miRNA species (Sledz et al., 2003; Hornung et al., 2005; Judge et al., 2005; Sioud, 2005; Reynolds et al., 2006). A variety of proteins, commonly referred to as pattern recognition receptors (PRRs), act as cellular sensors of exogenous RNA both extra- and intracellularly, where they monitor predefined hallmarks of foreign, typically viral, RNA such as length, 5′-end triphosphates, sequence composition, helical structure, and end-groups (Sioud, 2009). Most important to siRNA recognition are three members of the transmembrane Toll-like Receptor (TLR) family, namely TLR3 and TLR7/8, which recognize double or single-stranded RNA, respectively, primarily in immune cells and endo and epithelial linings of the body, as described below.

### Detection of siRNAs by TLR3

TLR3 senses the length of exogenous dsRNA (Alexopoulou et al., 2001) on the cell surface and in endosomes of dendritic cells, epi-, and endothelial linings (Cario and Podolsky, 2000; Kleinman et al., 2008; Lim et al., 2009; Zimmer et al., 2011; Taura et al., 2012) and consequently in many primary cells and popular cell lines (Kariko et al., 2004; Reynolds et al., 2006). Twenty-three mer siRNAs were early reported to trigger low-level immunogenicity through TLR3 in HEK293 cells (Kariko et al., 2004), an effect that was subsequently found highly duplex length and cell type specific; Reynolds et al. (2006) found that canonical 21-mer siRNAs did not trigger innate immune responses in human cell lines whereas longer siRNAs (23–33 mer) led to concentration-dependent, sequence-independent and presumably TRL3 mediated interferon-responses in some cell lines (DU145, HeLa S3, and MCF-7), but not in other cell lines (HeLa and Hek293 cells). In agreement, a very comprehensive study shows that longer DsiRNAs are indeed more immunogenic than 21-mer siRNAs both *in vitro* and *in vivo* (Foster et al., 2012) and that such dsiRNA designs are therefore not suited for siRNA therapeutics in a chemically non-modified form. Notably, sequence-independent activation of surface-bound TLR3 on retinal pigmented epithelial cells has been reported in a murine choroidal neovascularization model upon intraocular injection of naked 21-mer siRNAs (Kleinman et al., 2008). Also, shorter 19-mer siRNA designs did not trigger TLR3 activation, observations that were subsequently confirmed in other mouse models (Cho et al., 2009; Berge et al., 2010).

### Preventing activation of TLR3

The activation of murine TLR3 by unformulated 21-mer siRNAs has raised natural concerns for future siRNA therapeutics. However, similar effects may not apply to human cells; Weber et al. (2012) found that none of the tested TLR3 expressing human cell lines were responsive to unformulated, naked 21-mer siRNA, which may be due to sequence differences in the RNA binding ectodomain among species (Ranjith-Kumar et al., 2007). Still, researches may choose to use only 19-mer siRNA design that seem non-immunogenic and accept the slightly reduced potency of this siRNA platform or may instead choose to chemically modify the 21-mer siRNA structure to make it unrecognizable to TLR3, a task that is currently ongoing. In this regard, activation of PKR, a cytoplasmic dsRNA sensor, has been abrogated in HeLa cells by site-specific introduction of purine N2-benzyl modifications in the siRNA passenger strand (Puthenveetil et al., 2006). Also, disrupting the structure of a 47-bp dsRNA by introduction of GU wobble base pairs or introduction of 2′-deoxyuridine, 4-thiouridine, and 2-thiouridine (s2U) reduced PKR activation whereas 2′-F and PS did not (Nallagatla and Bevilacqua, 2008). Such abrogation of PKR activation suggests that chemical modification may similarly help reduce potential TLR3 activation by 21-mer siRNA.

### Detection of siRNA by endosomal TLR7/8

Small interfering RNAs can, upon internalization by endocytosis, be recognized by TLRs 7 and 8 (TLR7/8), which are transmembrane receptors exclusively found in the endosomes in different leukocyte subsets; TLR7 is mainly expressed in plasmacytoid dendritic cells (pDCs) and B cells, whereas TLR8 is expressed in monocyte-derived DCs (mDCs), monocytes, macrophages, or regulatory T cells (Hornung et al., 2002; Heil et al., 2004; Kokkinopoulos et al., 2005). TLR7/8 are reported to recognize specific single-stranded RNA motifs, which may be exposed from the siRNA duplex upon random thermal fluctuations. A number of immune-stimulatory GU- or U-rich motifs can trigger TLR7 activation such as GUCCUUCAA (Judge et al., 2005), UGUGU (Sioud, 2006), UGGC (Fedorov et al., 2006), UCA (Jurk et al., 2011), and GU (Diebold et al., 2004; Heil et al., 2004) whereas AU-rich motif primarily stimulate TLR8 (Forsbach et al., 2008). Collectively, such motif all agrees with the consensus that multiple uridines in close proximity are both necessary and sufficient for TLR7 stimulation (Diebold et al., 2006) whereas the exact sequence requirement and positional effects within the siRNA are not fully understood (Gantier et al., 2008; Goodchild et al., 2009); In fact, a single uridine may be sufficient to activate an immune response in a 5′-UCA-3′ sequence context whereas other uridine positions are non-immunogenic (Jurk et al., 2011).

### Abrogating TLR7/8 activation by chemical modification

Activation of endosomal TLR7/8 is currently considered the major source of siRNA immunogenicity *in vivo* (Hornung et al., 2005; Judge et al., 2005; Sioud, 2005; Cekaite et al., 2007; Robbins et al., 2008; Zamanian-Daryoush et al., 2008), which suggest that most siRNA immunogenicity may be avoided in human cells either by using delivery method that prevents siRNA endosomal retention (Sioud, 2005; Inaba et al., 2012) or by rendering siRNAs unrecognizable to TLR7/8 by chemical modification. The latter has been achieved by modifying the specific immunogenic sequences within the siRNA duplex; typically, small 2′ OH substitution have been employed, such as 2′-F, 2′-H, and 2′-OMe, as these can effectively abrogate TLR interaction while generally preserving silencing activity even upon extensive modification. Most efforts have focused on modifying U’s or U-rich regions within either or both strands of the siRNA (Heil et al., 2004; Diebold et al., 2006) as uridine residues are critical for siRNA activation of TLR7/8 (Heil et al., 2004; Hornung et al., 2005; Diebold et al., 2006; Eberle et al., 2008) and a significant correlation between the U count and siRNA immunogenicity has been suggested (Diebold et al., 2006; Goodchild et al., 2009). In fact, the rather commonly used UU overhangs can, in some cases, contribute to the activation of TLR7/8 (Goodchild et al., 2009). Modification of siRNA uridines with either 2′-F or 2′-OMe (Cekaite et al., 2007) or deoxynucleotide residues (Eberle et al., 2008) has been suggested as a general strategy to abrogate siRNA immunogenicity while preserving siRNA potency; In this regard, 2′-F is slightly less efficient in reducing siRNA immunogenicity than, e.g., 2′-OMe, which conversely is more likely to reduce siRNA potency (Prakash et al., 2005; Shin et al., 2007; Fucini et al., 2012). Notably, not only uridine, but also guanidine and adenosine modification have been reported to reduce siRNA immunogenicity, at least when a certain threshold of modification is met, whereas cytidine modifications have no effect (Hornung et al., 2005; Kariko et al., 2005; Morrissey et al., 2005b; Judge et al., 2006; Eberle et al., 2008; Fucini et al., 2012; Ocampo et al., 2012). Recently, selective adenosine modification, especially by 2′-OMe, has been recommended as it was found to most consistently reduce immunogenicity upon both 2′-OMe and 2′-F modification of different sequences (Robbins et al., 2007; Fucini et al., 2012). Encouragingly, 2′-OMe is particular potent in reducing siRNA immunogenicity (Robbins et al., 2007; Fucini et al., 2012) and alternating 2′-OMe modification of the passenger strand has been proposed as a universal approach to avoid TLR7 activation as it does not reduce guide strand potency (Hamm et al., 2009). This special property of 2′-OMe-modified RNA may reflect it being a potent antagonist of TLR7 activation: even single 2′-OMe modification can reduce immunogenicity *in vitro* and *in vivo* (Tluk et al., 2009) and 2′-OMe-modified oligoes can reduce the immunogenicity of other RNAs in *trans* upon co-transfection (Robbins et al., 2007).

The strength of hybridization between the siRNA strands has been suggested to correlate negatively with immunogenicity; interspersed CG or GC clamps may inhibit TLR7/8 activation by increasing siRNA thermostability and thereby impair the exposure of stimulatory single-stranded RNA upon breathing (Goodchild et al., 2009). Indeed, enhancing siRNA thermostability, e.g., by moderate LNA modification even of the strand opposing the putative immunogenic motif (Hornung et al., 2005; Goodchild et al., 2009; Schyth et al., 2012), may reduce siRNA immunogenicity by making the single-stranded immunogenic sequence-motif inaccessible to TLR7/8 recognition.

Other modifications types have also been shown to reduce TLR7/8 activation such as backbone PS modifications (Jurk et al., 2011) and base modifications including 5-methylcytidine (m5C), 5-methyluracil (m5U), N6-methyladenosine (m6A), s2U, or pseudouridine (Kariko et al., 2005) but are not widely used due to the success of other modification types, particularly 2′-OMe.

### Sensors of cytoplasmic siRNA

Cytoplasmic siRNAs, originating from endosomal release or direct cytoplasmic transfection, are monitored by a number of cytoplasmic PRRs that, similarly to the TLRs, respond to predefined RNA characteristics. A prominent sensor of dsRNA length is the dsRNA-dependent protein kinase R (PKR) that was originally described to sequence-independently act on dsRNA longer than 30 bp for regulation of cytokine expression in most cells types (Manche et al., 1992). Later studies demonstrated that only 17 bp dsRNA could activate PKR signal (Nanduri et al., 1998) and indeed modest PKR activation by 21-mer siRNAs has been reported in, e.g., microglial N9 (Zhang et al., 2006), T98G (Sledz et al., 2003; Marques et al., 2006; Puthenveetil et al., 2006), HeLa (Puthenveetil et al., 2006), MCF-7 (Armstrong et al., 2008), and HepG2.2.15 cells (Han et al., 2011). RIG-I is another prominent sensor of cytoplasmic dsRNA that can recognize dsRNA as small as 25bp and 5′-end triphosphates in a sequence-independent manner (Marques et al., 2006; Takahasi et al., 2008). The canonical 21-mer siRNA design having two 2 nt 3′ overhangs is tolerated, yet blunt 21–27-mer siRNA can activate RIG-I (Marques et al., 2006). Also, melanoma differentiation-associated gene 5 (Mda5) has been reported to respond to siRNA (Kang et al., 2002).

The impact of modest cytoplasmic PRR activation needs to be studied further, a task that is complicated by the complex interplay of signaling pathways during innate immune responses. Notably, direct cytoplasmic delivery of siRNA by electroporation into human blood cells abrogated the siRNA immunogenicity observed when delivering identical siRNAs via endosomes using lipids, which suggests that synthetic siRNAs are rather detected by TLRs than cytoplasmic sensors in immune cells (Sioud, 2005). Therefore researches tend to focus more on reducing the potent TLR-mediated responses during application *in vivo*. Still, PKR activation can be reduced by chemical modification such as N2-benzyl modifications (Puthenveetil et al., 2006) 2′-deoxyuridine, 4-thiouridine, and s2U (Nallagatla and Bevilacqua, 2008) as stated above. The low RIG-I activity seen when using 21-mer siRNA with canonical 2 nt 3′-overhang has left this pathway less studied, however, blunt-ended siRNAs, such as some asymmetric dsiRNAs designs, may be modified by 2′-OMe (Collingwood et al., 2008; Zamanian-Daryoush et al., 2008) or DNA (Marques et al., 2006) to reduce RIG-I activation.

## Reducing siRNA Off-Target Effects by Chemical Modification

The high sequence specificity of siRNA action was immediately praised as a major benefit of RNAi-based gene silencing. Indeed, The RISC/Ago2-mediated cleavage of target RNAs by a matching siRNA is highly sequence-specific allowing only few mismatches, particularly at siRNA terminal ends (Du et al., 2005; Dahlgren et al., 2008). Hereby cleavage of unintended targets can simply be avoided during siRNA design by BLASTing candidate sequences against the relevant transcriptome. Still, siRNA significantly influence the target cell transcriptome in a sequence and concentration-dependent manner by triggering modest and unintended silencing of hundreds of endogenous genes (Doench et al., 2003; Jackson et al., 2003; Lim et al., 2005; Birmingham et al., 2006). Such so-called off-target effects may immediately remain unnoticed, but can lead to cellular toxicity (Fedorov et al., 2006), misinterpretation of an RNAi experiment (Lin et al., 2005), and are unacceptable in a therapeutic setting.

Off-target effects reflect the shared handling of siRNA and miRNA by the RNAi pathway, which inherently enable siRNAs to behave as miRNAs (Doench et al., 2003). Hereby siRNA off-targeting is primarily mediated by the interaction between the seed region of the RISC-associated guide strand (positions 2–7/8 counting from the 5′-end) and complementary sequences in mRNA 3′-UTRs (Lin et al., 2005; Birmingham et al., 2006) leading to translational inhibition and mRNA destabilization rather than mRNA cleavage by Ago2 (Doench and Sharp, 2004; Wu et al., 2006; Baek et al., 2008; Guo et al., 2010). Although off-target effects are somewhat predictable and may be minimized by *in silico* analysis (similar to miRNA target prediction) they cannot be fully avoided and remain an inherent feature of unmodified siRNAs (Doench et al., 2003; Jackson et al., 2006b). Off-target effects can be minimized by using the lowest possible dose of siRNAs sufficient for efficient target KD (Jackson et al., 2003, 2006b) or by using siRNA pools directed against multiple target sites in the same mRNA to reduce the contribution of individual siRNAs to off-targeting (Myers and Ferrell, 2005; Myers et al., 2006). Yet, the competition between co-transfected siRNAs for incorporation into RISC complicates this latter strategy (Castanotto et al., 2007). Furthermore, as off-targeting can be mediated by both strands of the duplex (Wei et al., 2009), designing siRNA with optimal thermodynamic asymmetry will ensure preferential guide strand uptake into RISC and reduce passenger strand off-targeting.

A more indirect type off-targeting comes from the competition between exogenous siRNA and endogenous miRNAs for RNAi proteins; siRNA (and miRNAs) have different affinities for RISC (Koller et al., 2006; Castanotto et al., 2007; Vickers et al., 2007), which is reportedly determined by the siRNA seed sequence (Yoo et al., 2008) and siRNAs can effectively compete out endogenous miRNA leading to deregulated gene expression (Khan et al., 2009). Also, thermodynamically stable siRNAs may be more slowly processed by the RNAi machinery leading to clotting of the RNAi pathway and natural gene regulation.

### Minimizing passenger strand off-targeting by chemical modification

Owing to the structural symmetry of the canonical siRNA, both strands may potentially contribute to off-targeting (Wei et al., 2009), a matter that has been resolved by chemical modification. For siRNAs designed with insufficient thermodynamic asymmetry between duplex ends, selective unwinding of the siRNA from the 5′-end of the guide strand can be favored by introducing additional asymmetric thermostability by chemical modification identical to those that aim to enhance siRNA potency through selective guide strand loading. Similarly, asymmetric chemical modification of siRNA overhangs can disfavor passenger strand selection during RISC loading to enhance silencing specificity (see Designing Potent siRNAs by Chemical Engineering). Chemical modification may also be employed to fully prevent loading of the passenger strand: The 5′-phosphate of the passenger strand, required for strand function (Nykanen et al., 2001), may be chemically blocked, e.g., by 5′-OMe modification to prevent binding the AGO MID domain (Chen et al., 2008). Similarly, a single UNA modification at the 5′-terminus of the passenger strand can reduce unspecific passenger strand activity (Vaish et al., 2011).

A radical approach to prevent passenger strand off-targeting is to use alternative siRNA designs; aiRNAs or asiRNAs habour shortened passenger strands which cannot be efficiently loaded into RISC (Chu and Rana, 2008; Sun et al., 2008; Chang et al., 2009) whereas the three stranded sisiRNA design renders the passenger strand non-functional by splitting it into two shorter strands incapable of directing RNAi (Bramsen et al., 2007).

### Reducing off-target effects by modifying the guide strand seed region

The initial interaction between the siRNA guide strand (or miRNA) and target RNA is mediated by the seed region exposed by RISC (Wang et al., 2008). Therefore, mismatches in or chemical modification of this region have a severe effect on the miRNA-like interference that often relies only on seed matching (Birmingham et al., 2006) whereas fully matched siRNAs can usually accommodate a single mismatch at selected seed positions without a substantial loss of silencing efficacy (Du et al., 2005; Dahlgren et al., 2008). Therefore position-specific modifications in the seed region of the guide strand have been screened to reduce the siRNA off-targeting potential while preserving siRNA effects though target cleavage. Jackson et al. (2006a) demonstrated that 2′-OMe modification can site-specifically reduce off-targeting when positioned at guide strand position 2 without severe reductions in siRNA potencies. A similar screen reported that substituting the entire guide strand seed region, e.g., positions 1–8 with DNA could also reduce off-targeting although the effects seemed more sequence-specific (Ui-Tei et al., 2008). Recently, we showed that incorporation of the strongly destabilizing UNA modification at position 7 very potently eliminated off-target effects with minimal negative impact on siRNA efficiency in three different siRNA tested (Bramsen et al., 2010), a result subsequently confirmed by others (Vaish et al., 2011). Although these finding greatly enhance the therapeutic potential of siRNAs, it remains to be fully explored how applicable these modification principles are translated to RNAi *in vivo*.

### Alternative means to reduce off-targeting

Off-targeting by siRNA may also be reduced by insuring that siRNAs are primarily loaded into RISC complexes harboring the cleavage-competent Ago2 in contrast to Ago1, -3, or -4-containing RISC, which would not allow target cleavage, but merely miRNA-like effects. Synthetic siRNA duplexes interact with all Ago proteins, yet the non-catalytic Ago1, -3, and -4 can be selectively inhibited by stabilizing siRNA with LNA hereby leading to increased silencing activity via Ago2-RISC and potentially less off-targeting (Petri et al., 2011). Also, the perturbance of endogenous miRNA pathways upon siRNA transfection may quite naturally be minimized by using a minimal amount of siRNAs (Khan et al., 2009). Alternatively, chemical modification has been shown to influence siRNA competitive powers (Yoo et al., 2008) and chemical modification schemes that ensure sufficient silencing with a minimum of miRNA pathway perturbance are likely to be established soon.

## Final Remarks: Building and Making Good Use of Chemically Optimized siRNAs

There is little doubt that chemical engineering can bring siRNAs closer to clinical success and, as explored throughout this text, it is now fairly well established how chemical modification can enhance siRNA potency, nuclease resistance, and reduce siRNA immunogenicity and off-targeting. However, the difficulty of efficient and targeted siRNA delivery *in vivo* represents the major bottleneck for broader clinical application and concurrently hampers the evaluation and further chemical refinement of siRNA performance and it seems that more immediate clinical success is pivotal to maintaining the high momentum in siRNA drug development. In this regard, implementation of promising local delivery systems by smaller RNAi R&D companies may prove most promising, while universal systemic delivery strategies, commercialized by major Pharma industries, are more distant goals.

Very extensively or fully modified siRNAs have typically been most successful *in vivo* during early development of chemically modified siRNAs (Soutschek et al., 2004) but may also be inhibitory to rapid advances into clinical testing due to higher cost and availability of the modified nucleotides. However, the initial reports from clinical application suggests that high modification levels may not always be needed, even for unformulated siRNAs (Leachman et al., 2010), and the concurrent use of protective siRNA delivery vehicles predicts that siRNA modification levels may be kept relatively low. While awaiting more clinical success of siRNA therapeutics our knowledge of natural gene silencing pathways is expanding and particularly the regulation and deregulation of miRNAs expression in human diseases hold diagnostic, prognostic and therapeutic value. Strategies for replenishing reduced or lost miRNA expression in human disease by introducing miRNA mimics or inhibiting aberrantly expressed miRNAs via synthetic anti-miRs is currently gaining momentum (Bader et al., 2010). As synthetic siRNA and miRNA mimic are practically identical, the huge arsenal of chemical modification schemes developed for siRNAs will greatly aid the fast development of synthetic miRNA therapeutics in the years to come.

### A quick guide to chemical siRNA design

**Figure 4:**
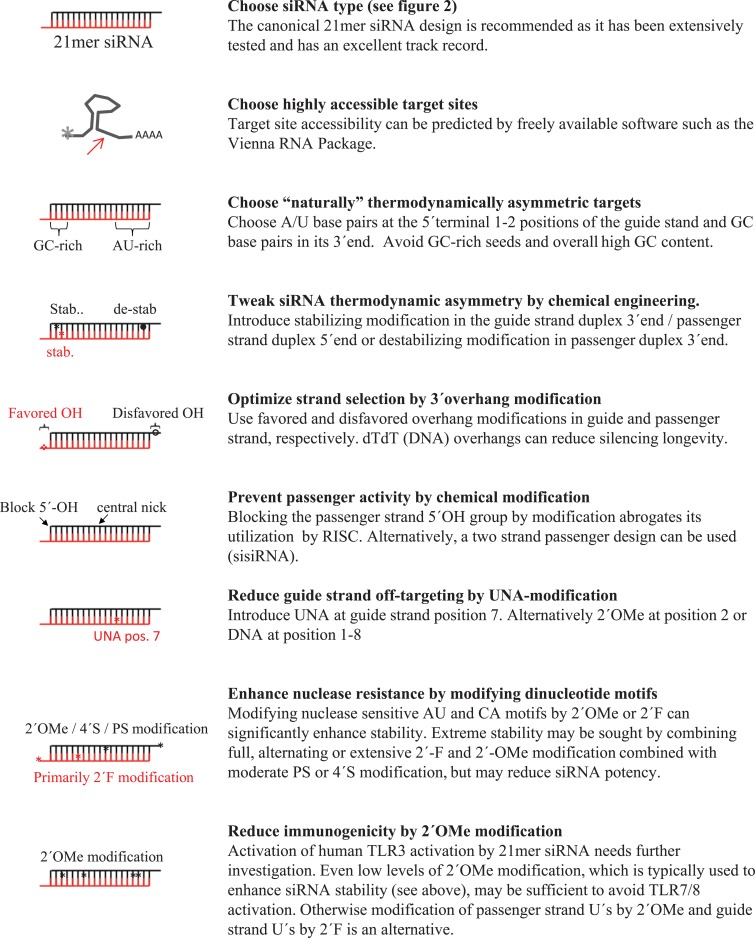
**Suggested siRNA design scheme**. Please see text for details.

In Figure 4 we summarize our own general strategy for siRNA design and modification, which aim to keep modification levels low. Whereas this may serve as an inspiration to fellow siRNA researchers, experimental validation of function *in vivo* is still needed and one may still consider utilizing more extensive and well-characterized modification schemes (as described in the text above).

We recommend that siRNA should be designed by first selecting the desired siRNA type; here the canonical 21-mer siRNA has been extensively tested and has produced potent and safe gene silencing *in vivo* although the concerning TLR3 activation observed in mice may need further evaluation in human cells. The primary siRNA sequence should be chosen to target highly accessible regions within the target RNA as the siRNA thermo-stability profile can subsequently be tweaked by chemical modification, whereas no modification types have been reported to improve siRNA performance on poorly accessible target. To optimize specificity, siRNA should not share extensive sequence complementarity to non-intended targets and off-targets base-pairing to siRNA positions 2–16 should preferentially be avoided. siRNAs should be designed to be thermodynamically asymmetric, preferably by primary sequence selection of A/U base-paring at positions 1–2 and G/C base-pairing at position 19 of the guide strand. To introduce additional thermodynamic asymmetry, destabilizing modification can be introduced in the passenger 3′-end or stabilizing modification in the 5′-end, if needed. Similarly, asymmetric overhang modification can affect strand selection to enhance siRNA potency and specificity and modestly enhance nuclease resistance, whereas dTdT overhangs should be avoided as they reduce silencing longevity. Also, the incorporation of the passenger strand into RISC can be fully avoided by blocking its 5′-phosphate group. Importantly, miRNA-like off-targeting is best reduced by introducing UNA at position 7 of the guide strand, but 2′-OMe and DNA modification of the guide strand seed have also proved efficient. siRNA immunogenicity and nuclease resistance may likely be simultaneously addressed by chemical modification with 2′-OMe and 2′-F, possibly in combination with limited PS backbone or 4′-Thio modification. As 2′-OMe modification may fully abrogate TLR7/8 responses, nuclease sensitive sites (primarily UA and CA) may be modified by 2′-OMe in the passenger strand to enhance siRNA stability while abrogating immunogenicity. As 2′-OMe may not be well-tolerated in the guide strand seed, 2′-F modification should instead be used at nuclease sensitive sites in this region. Alternatively, both nuclease resistance and immunogenicity may be addressable by slightly enhancing siRNA thermodynamic stability, e.g., by LNA modifications.

## Conflict of Interest Statement

The authors declare that the research was conducted in the absence of any commercial or financial relationships that could be construed as a potential conflict of interest.
